# Drug Discovery of Host CLK1 Inhibitors for Influenza Treatment

**DOI:** 10.3390/molecules201119653

**Published:** 2015-11-02

**Authors:** Mian Zu, Chao Li, Jian-Song Fang, Wen-Wen Lian, Ai-Lin Liu, Li-Shu Zheng, Guan-Hua Du

**Affiliations:** 1 Institute of Materia Medica, Chinese Academy of Medical Sciences & Peking Union Medical College, Beijing 100050, China; zumian@ihep.ac.cn (M.Z.); lichao880919@imm.ac.cn (C.L.); fangjiansong0815@163.com (J.-S.F.); lianwenwen1989@imm.ac.cn (W.-W.L.); 2 CAS Key Laboratory for Biomedical Effects of Nanomaterials and Nanosafety, Institute of High Energy Physics, Chinese Academy of Sciences, Beijing 100049, China; 3 Beijing Key Laboratory of Drug Target Research and New Drug Screening, Beijing 100050, China; 4 State Key Laboratory of Bioactive Substance and Function of Natural Medicines, Beijing 100050, China; 5 National Institute for Viral Disease Control and Prevention, Chinese Center for Disease Control and Prevention, Beijing 102206, China

**Keywords:** influenza A virus, CLK1 inhibitor, CPE assay, baculovirus coinfection, protein expression and purification, rational screening

## Abstract

The rapid evolution of influenza virus makes antiviral drugs less effective, which is considered to be a major bottleneck in antiviral therapy. The key proteins in the host cells, which are related with the replication cycle of influenza virus, are regarded as potential drug targets due to their distinct advantage of lack of evolution and drug resistance. Cdc2-like kinase 1 (CLK1) in the host cells is responsible for alternative splicing of the M2 gene of influenza virus during influenza infection and replication. In this study, we carried out baculovirus-mediated expression and purification of CLK1 and established a reliable screening assay for CLK1 inhibitors. After a virtual screening of CLK1 inhibitors was performed, the activities of the selected compounds were evaluated. Finally, several compounds with strong inhibitory activity against CLK1 were discovered and their *in vitro* anti-influenza virus activities were validated using a cytopathic effect (CPE) reduction assay. The assay results showed that clypearin, corilagin, and pinosylvine were the most potential anti-influenza virus compounds as CLK1 inhibitors among the compounds tested. These findings will provide important information for new drug design and development in influenza treatment, and CLK1 may be a potent drug target for anti-influenza drug screening and discovery.

## 1. Introduction

Influenza A viruses are enveloped negative-sense, single-stranded segmented RNA viruses belonging to a genus of the Orthomyxoviridae family of viruses, which cause influenza in birds and some mammals. Several subtypes are labeled according to their number of glycoproteins neuraminidase (NA) and hemagglutinin (HA) [1]. Influenza A viruses pose an important global health threat that causes up to approximately 30,000 deaths and 200,000 hospitalization every year, respectively. Besides, the average global burden of inter-pandemic influenza leads to more than 300,000–500,000 deaths worldwide, and on the order of 1 billion cases per year [2]. Up to now, amantadine and rimantadine, oseltamivir and zanamivir, as two classes of drugs approved by the FDA, are designed to target the viral proteins M2 ion-channel and neuraminidase, respectively. However, owing to the high resistance rate of influenza virus, the former drugs are not recommended as treatment options for anti-influenza therapy [3]. Though the current circulating H1N1, H3N2, and Flu B strains are largely sensitive to anti-NA drugs, the application of the latter has been limited during the recent widespread seasonal epidemics [4]. This situation raises concern for the research and development of antiviral drugs with new targets for flu treatment.

As an alternative therapeutic strategy, targeting host cell cofactors which play a key role in the influenza virus replication cycle may greatly reduce the possibility of emergence of viral resistance. The cdc2-like kinases (CLKs) are an evolutionarily conserved kinases with dual specificity. The CLK family consists of four isoforms: CLK1, CLK2, CLK3 and CLK4. CLK1 play an important role in the regulation of alternative splicing of human genes through multisite phosphorylation of their RS serine/arginine-rich (SR) family of splicing factors [5]. Some key technologies such as genome-wide RNA interference (RNAi) screening have accelerated the discovery and identification of host factors [6,7]. As one of these host cofactors which have been identified by RNAi required for influenza virus replication, CLK1 regulates alternative splicing of viral M1 pre-mRNA into M2 mRNA by phosphorylating the spliced factor SF2/ASF in infected primary normal human bronchial epithelial (NHBE) and human lung epithelial (A549) cellular assays. Moreover, further studies showed that TG003, a small molecule inhibitor of CLK1, could reduce influenza virus replication and impair the splicing of viral M2 mRNA [8,9]. Therefore, CLK1 may perform as a potent target for novel antiviral drug discovery. 

Although several CLK1 inhibitors have been reported, including the benzothiazole compound TG003 (IC_50_, 10 nM for CLK1) [10,11], the quinazoline NCGC00010037 (IC_50_, 37 nM for CLK1) [12], the indole KH-CB19 (IC_50_, 20 nM for CLK1) [13], and some flavonoid derivatives [14], little is known about their inhibitory effects on influenza virus replication. In this study, the recombinant human CLK1 protein was successfully expressed and purified, then a screening assay for CLK1 inhibitors was set up and used for the discovery of new CLK1 inhibitors. Virtual screening was applied in this study before entity screening, and the compound library used in the study is an in-house database, which belongs to the National Center for Pharmaceutical Screening, Institute of Materia Medica, Chinese Academy of Medical Sciences (Beijing, China). After rational screening, several CLK1 inhibitors were found. Finally the *in vitro* anti-influenza virus activities of the CLK1 inhibitors were evaluated by the cytopathic effect assay to explain the possibility of using CLK1 as the antiviral drug target. 

## 2. Results

### 2.1. Construction of Recombined CLK1 Baculovirus, Protein Expression, Identification and Purification 

**Figure 1:**
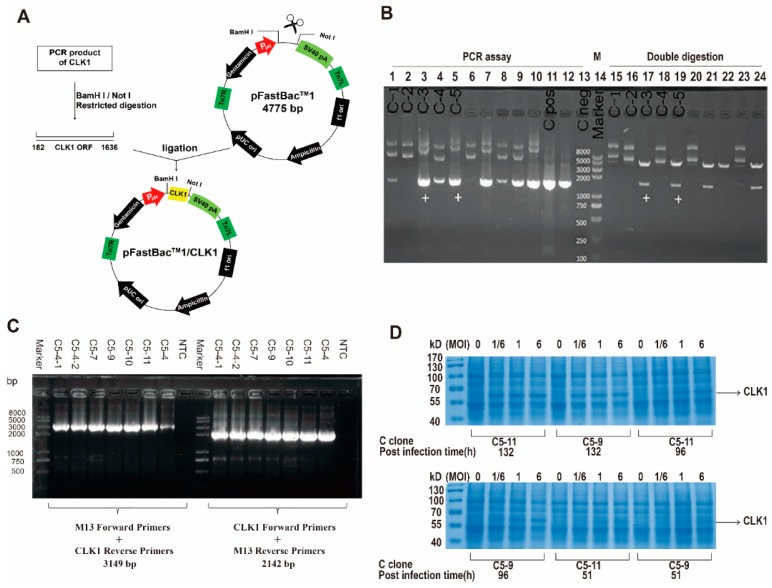
Construction of CLK1/pFastBac1 plasmid and the protein expression of CLK1 in insect cells. (**A**) A schematic of the steps to construct CLK1/pFastBac1 recombinant plasmid; (**B**) Identification of the constructed CLK1/pFastBac1 recombinant plasmid by both colony PCR assay and double digestion. C-1~5: clone 1 to 5. Cpos: positive clone. Cneg: negative clone; (**C**) Identification of recombinant bacmid CLK1/pFastBac1 by PCR analysis. NTC: non-template control; (**D**) Determination the optimal conditions to express the recombinant protein His_6_-CLK1 by Coomassie Brilliant Blue staining.

**Figure 2:**
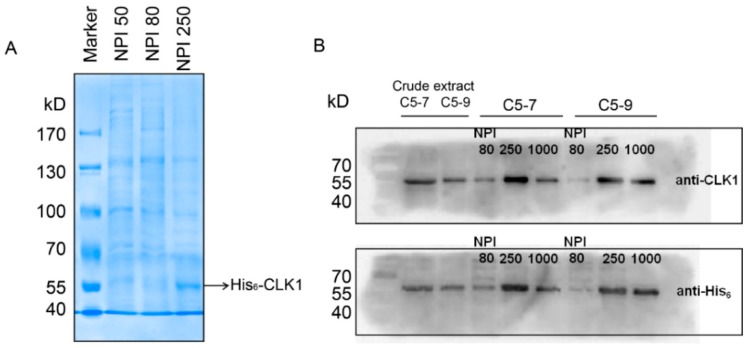
Analysis of CLK1 recombinant protein purified by Ni-NTA column. (**A**) SDS-PAGE and Coomassie staining of purified His_6_-CLK1 by Ni-NTA column after baculovirus-mediated expression in Sf9 insect cells. NPI50, NPI80 and NPI250 represent the increasing concentrations of imidazole in the elution buffer are 50, 80 and 250 mM, respectively. NPI250 shows purified His_6_-CLK1 subsequently eluted from the Ni-NTA affinity column; (**B**) Western Blot analysis of CLK1 and His in crude insect cellular extract and purified His_6_-CLK1 components.

**Figure 3:**
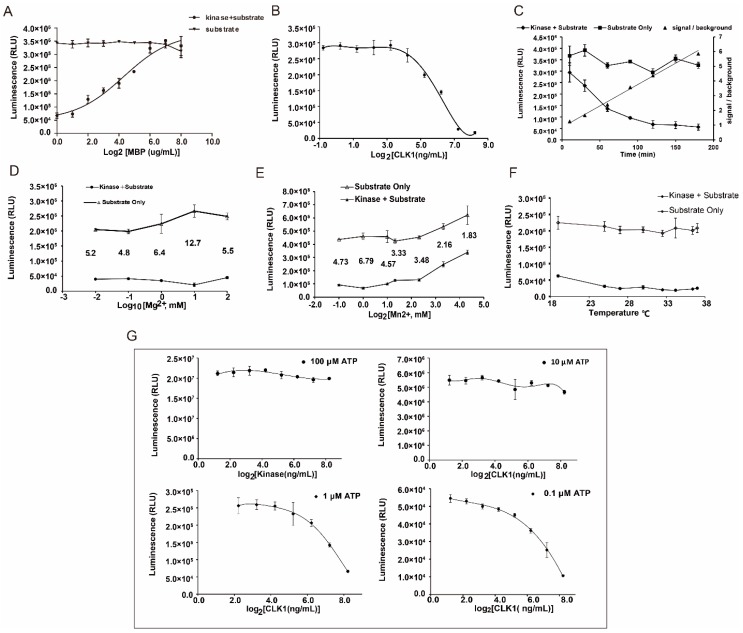
Optimization of parameters in Kinase-Glo^®^ luminescent kinase assay. (**A**) Substrate concentration-dependent phosphorylation of CLK1; (**B**) Enzyme concentration-dependent phosphorylation of CLK1. ATP concentrations and MBP concentrations were 1 μM and 50 ng/μL, and incubation times were 90 min at 25 °C; (**C**) Time-dependent phosphorylation of CLK1. The incubation times of kinase reaction were set as 10, 30, 60, 90, 120, 150 and 180 min; (**D**) Mg^2+^ concentration-dependent phosphorylation of CLK1; (**E**) Mn^2+^ concentration-dependent kinase reaction of CLK1; (**F**) Temperature-dependent phosphorylation of CLK1; (**G**) ATP concentration-dependent phosphorylation of CLK1 with concentrations of 0.1, 1, 10 and 100 μM.

**Figure 4:**
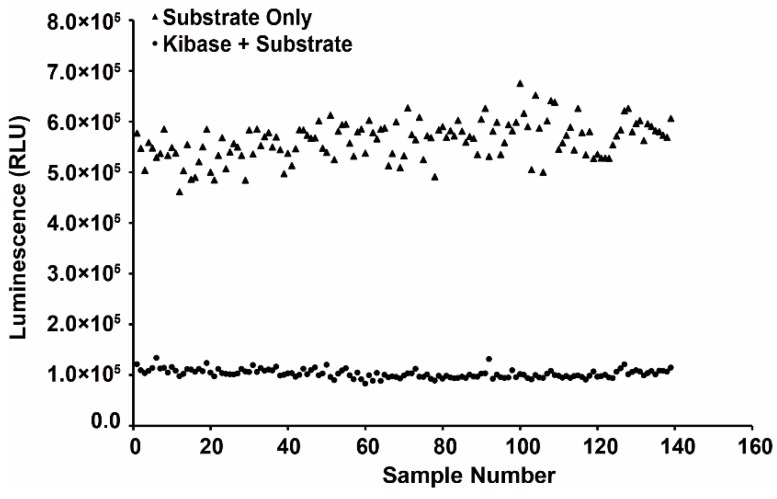
Evaluation of the screening model for CLK1 inhibitor. The calculated Z′ value was 0.70. RLU = relative light unit.

**Figure 5:**
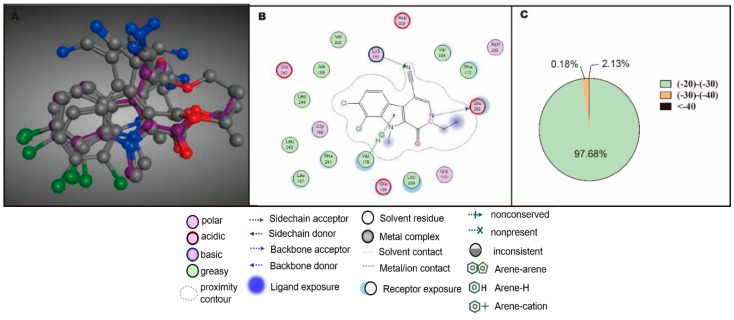
Virtual screening of lead compounds with CLK1 inhibitory effect. (**A**) Top five poses docking of ligand V25 in binding pocket of 2VAG, with RMSD value of 1.38 Å; (**B**) The interaction between ligand V25 and receptor is crucial for binding; (**C**) The proportion of different levels of scores according to virtual screening.

The construction procedure was performed according to Figure 1A. The total RNA extracted from Human Umbilical Vein Endothelial Cells (HUVEC) was used to synthesize cDNA, which was performed as the template in PCR to produce full-length coding sequence (CDS) of CLK1 gene with *BamH* I and *Not* I at both ends, respectively. After the ligation reaction, the recombined CLK1/pFastBac1 plasmid was transformed into DH5α *E. coli* strain and five ampicillin-resistant transformants were selected as follows: C-1, C-2, C-3, C-4 and C-5. As shown in Figure 1B, C-3 and C-5 were identified to be correct clones with correct insert orientation by colony polymerase chain reaction (PCR) assay and restriction analysis.

The purified plasmid C-5 was transformed into DH10Bac™ *E. coli* for the bacmid. 48 h after transformation, 10 white clones were picked and restreaked on fresh LB agar plate containing appropriate antibiotics. Then recombinant bacmid DNA was isolated and analyzed by PCR and gene sequencing to verify successful transposition to the bacmid. Finally C5-9 and C5-11 were selected in the following procedures (Figure 1C).

After high-titer P1 and P2 baculoviral stock were generated, insect Sf9 cells were transfected by P2 baculovirus stock for optimizing the protein expression. As shown in Figure 1D, compared with virus-free control, C5-9 and C5-11 with 1/6, 1 and 6 PFU per cell at 132 h post-infection or 6 PFU per cell at 96 h post-infection have the high expression level. Then His_6_-CLK1 was purified by immobilized metal-ion affinity chromatography. Cell lysate was applied to the column containing Ni-NTA matrices, followed by elution buffers with series concentrations of imidazole (20 mM, 50 mM, 80 mM and 250 mM) through the column. As is shown in Figure 2A, most of His_6_-CLK1 proteins with the molecular weight of 55 kD accumulates in elution buffer of NPI 250 containing 250 mM imidazole, rather than that of NPI50 or NPI80. Immunoblotting using a monoclonal antibody showed the same result (Figure 2B). The NPI250 fraction was concentrated and frozen in kinase storage buffer containing 20% glycerol for further analysis.

### 2.2. Establishment of Drug Screening Assay for CLK1 Inhibitors 

The optimal conditions were dependent on the largest change in luminescence when comparing kinase reaction wells with control wells. As is shown in Figure 3, myelin basic protein (MBP) at the concentration of 0.05 μg/μL, CLK1 at 149 ng/mL, ATP at 1 μM, Mg^2+^ at 10 mM, Mn^2+^ at 1 mM in 20 μL with the temperature of 25 °C for 90 min incubation were considered to be the optimal conditions of CLK1 inhibitory assay.

The stability of the assay should be evaluated before application. A series of validation samples which including 139 positive and other 139 negative controls was performed to calculate Z′ factor. As a result shown in Figure 4, the average luminescence (RLU) value of each well from positive control samples was 102,684 ± 8512, while the RLU value from negative ones was 563,010 ± 37,414, Hence the Z′ factor was 0.70 by estimation based on those RLU values above, which represented a satisfied level for a stable and suitable screening assay for CLK1 inhibitors.

As a known inhibitor of CLK1, TG003 was set as positive control in this assay. The IC_50_ value of TG003 was determined to be 8.1 nM, which was consistent with that of TG003 reported in the literature [10].

### 2.3. Rational Screening of CLK1 Inhibitors 

To reduce the cost and improve the drug discovery rate, virtual screening was performed. The docking program in MOE (Molecular Operating Environment, Version 2010.10, Chemical Computing Group, Quebec, QC, Canada) was used to assess the performance of each compound in the compound database with the active site of CLK1crystal structure 2VAG. The ligand in 2VAG was V25, the root-mean-square deviation (RMSD) value of the top five ligand V25 poses docking into the binding pocket of 2VAG was 1.38 Å (shown in Figure 5A), which identified the reliability of this docking method. The crucial interaction between ligand and receptor was also described as follows: H-bonding interaction between V25 and amino acid residues Lys191 and Glu292, conjugation effect between Val175-H and aromatic ring, and the hydrophobic interactions (shown in Figure 5B). The docking result was shown in Figure 5C, 2677 out of 21,758 compounds with docking scores were below −20 kcal/mol, 62 compounds with docking scores below −30 kcal/mol, accounting for 2.13%. The docking scores of the rest 97.68% compounds ranged from −20 to −30 kcal/mol.

**Figure 6:**
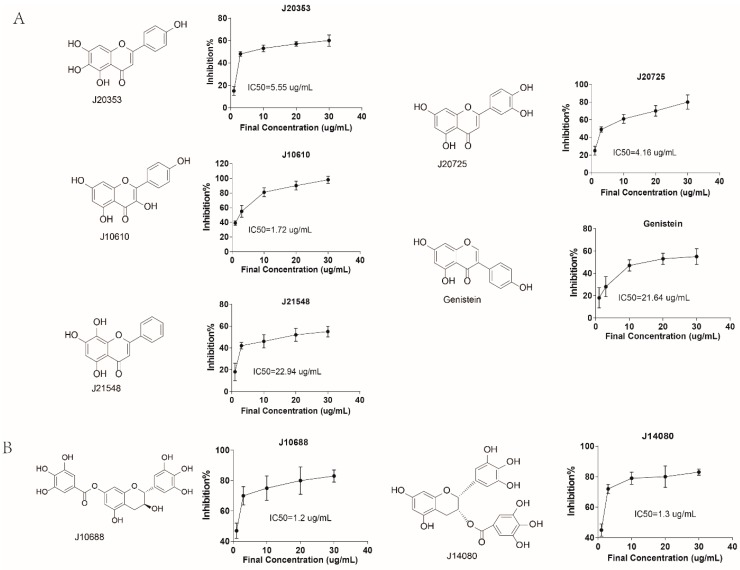

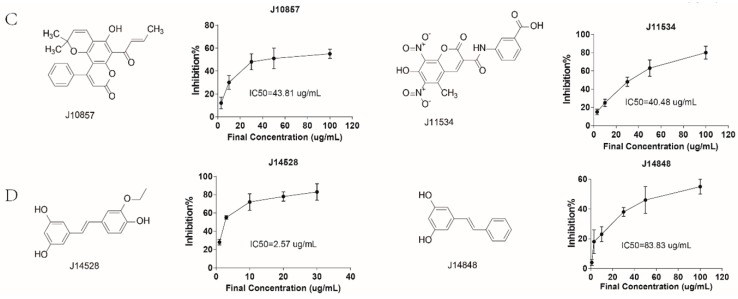
The structures and IC_50_s of CLK1 inhibitors after screening based on Kinase-Glo^®^ Assay. (**A**) flavonoids; (**B**) tannins; (**C**) coumarins; (**D**) stilbenes.

Additionally, 380 compounds out of 2677 compounds with better binding properties during virtual screening were selected to test their activities in 384-well plates using the kinase reaction system, with addition of 2 μL stock solution (100 μg/mL) of sample into each well, the total volume was 20 μL, while TG003 was set as the positive control. As shown in Figure 6, 15 compounds containing J20353, J10610, J20725, J21548, J25986, J12133, J12098, J14080, J10688, J10857, J11534, J14528, J14848, genistein and methyl gallate showed strong CLK1 inhibitory effects in a dose-dependent manner, with IC_50_s lower than 50 μg/mL. Among them, 10 compounds exhibited strong inhibitory effect with the IC_50_ value lower than 10 μg/mL and J12098 exhibited the best inhibitory effect, with IC_50_ value of 0.38 μg/mL. All of these 10 compounds were either natural products or natural product derivatives after structural modification (J11534 and J10857). Their structures can be divided into four types: flavonoids, tannins, coumarins and stilbenes. These active compounds had better docking results and showed closely interactions with receptor (Table S1).

### 2.4. In Vitro Anti-Influenza Viral Activities of CLK1 Inhibitors

Before cytopathic effect (CPE) reduction assay, cytotoxicity of all active compounds in Madin-Darby Canine Kidney (MDCK) cells were detected. The 50% cytotoxic concentration (CC_50_) and maximal non-cytotoxic concentration (CC_0_) are shown in Table 1.

From Table 1 we can see that most of the compounds had a relatively lower toxic except J14848 and J20725. CPE reduction assay was conducted to evaluate the anti-influenza ability of CLK1 inhibitors with three different drug administrations: simultaneous treatment assay, pre-treatment assay and post-treatment assay in MDCK cell. The results (Table 1) showed that most of CLK1 inhibitors exhibited antiviral effects in three administrations. Time of addition assay can preliminarily determine the way in which the compounds play the roles in virus infectious cycle. J12098, J14848 and J10857 exhibited similar functions in three kinds of administrations, indicating that these three compounds may act in the late stage of the virus replication. The 50% effective concentrations (EC_50s_) of J12133 and J20353 were 9.84 μg/mL and 5.26 μg/mL in simultaneous treatment assay, respectively, which meant they can inhibit virus interaction with cells and this way may be the main mechanism. Also, the two compounds performed better in pre-treatment assay than post-treatment assay, indicating direct effects to virus. J25986 and J20725 revealed significant difference in post-treatment assay from other two administrations and J25986 exhibited anti-viral effects in the three assays with EC_50_ of 3.42 μg/mL. It was inferred that they may influence the replication stage of the virus. The other two compounds, J10610 and J14080, had the lower EC_50s_ in the pre-treatment assay, which meant they can inhibit the virus directly. In conclusion, J10688 (clypearin), J12098 (corilagin) and J14848 (pinosylvin) were the most potential anti-influenza virus candidates as CLK1 inhibitors among the compounds.

**Table 1:** *In vitro* time-of-addition studies on anti-influenza virus activity of CLK1 inhibitors in MDCK cells by CPE reduction assay against H1N1.

Samples	Name	CC_50_^a^	CC_0_^b^	Pre-Treatment Assay		Simultaneous Treatment Assay		Post-Treatment Assay	
		μg/mL		EC_50_ (μg/mL)^c^	SI ^d^	EC_50_ (μg/mL)	SI	EC_50_ (μg/mL)	SI
J10688	Clypearin	>200	100	0.75 ± 0.13	>266.67	1.2 ± 0.28	>166.67	18.08 ± 3.02	>11.06
J12098	Corilagin	153.54	30	3.99 ± 3.72	38.48	2.00 ± 2.22	76.77	5.91 ± 1.44	25.98
J14848	Pinosylvin	18.26	10	7.54 ± 5.48	2.42	5.28 ± 2.45	3.46	6.65 ± 4.3	3.23
J12133	Chebulanin	90.27	30	15.29 ± 10.35	5.90	9.84 ± 0.44	9.17	21.11 ± 11.15	4.28
J25986	Propyl gallate	182.00	30	15.94 ± 6.94	11.42	12.88 ± 8.06	14.13	3.42 ± 0.2	53.22
J20353	Hispidulin	60.35	30	18.89 ± 5.1	3.19	5.26 ± 2.48	11.47	>30	ND
J21548	Norwogonin	91.79	30	>30	ND ^e^	>30	ND	>30	ND
J11534	-	175.31	30	>30	ND ^e^	>30	ND	>30	ND
J10610	Kaempferol	68.50	30	5.16 ± 2.4	13.28	12.34 ± 5.89	5.55	23.3 ± 1.08	2.94
J20725	Luteolin	24.86	10	>10	ND	>10	ND	5.74 ± 0.54	4.33
J10857	-	77.72	30	18.43 ± 12.99	4.22	16.27 ± 2.29	4.78	14.67 ± 5.25	5.30
J14528	Isorhapontigenin	83.37	30	5.98 ± 2.3	13.94	>30	ND	>30	ND
J14080	Epigallocatechin gallate	71.10	30	10.19 ± 6.22	6.98	14.86 ± 1.81	4.78	>30	ND
Methyl gallate	Methyl gallate	69.50	30	>30	ND ^e^	>30	ND	>30	ND
Genistein	Genistein	75.60	30	>30	ND	>30	ND	>30	ND
Ribavirin	Ribavirin	>200	100	4.36 ± 0.99	>45.87	10.32 ± 2.38	>19.38	13.78 ± 3.47	>14.51
TG003	TG003	121.50	30	5.09 ± 0.78	23.87	15.75 ± 3.49	7.71	3.56 ± 1.47	34.13

^a^ CC_50_: 50% cytotoxic concentration; ^b^ CC_0_: maximal non-cytotoxic concentration; ^c^ EC_50_: 50% effective concentration; ^d^ SI: selective index, CC_50_/EC_50_; ^e^ ND: not determined.

## 3. Experimental Section

### 3.1. Cell Culture

MDCK cells were maintained in Dulbecco’s modified Eagle medium (DMEM), which contained 10% fetal bovine serum (FBS) in 37 °C incubator filled with 5% carbon dioxide. The insect cell line Sf9 was cultured in SF-900™ II SFM complete growth medium at 27 °C in air.

### 3.2. Virus

The influenza virus A/PR/8/34 (H1N1) was kindly donated by the Institute for Viral Diseases Control and Prevention, Chinese Center for Disease Control and Prevention (Beijing, China). Viral stocks of these laboratory-adapted strains were propagated in 9-day-old embryonated chicken eggs for 48 or 72 h and stored at −80 °C. 

### 3.3. Compounds

TG003, purchased from Sigma Aldrich Company (Shanghai, China) was used as a reference compound in the CLK1 inhibitory assay. The compounds used for CLK1 inhibitors screening were from the sample library of the National Center of Pharmaceutical Screening, which were dissolved in dimethyl sulfoxide at 10 mg/mL as a stock solution.

### 3.4. Construction of the Recombinant Human CLK1 Baculovirus

The information of human CLK1 gene was obtained from Genebank (NCBI reference sequence: NM_004071.3). The primer of human full length CLK1 gene was as follows: CLK1-sense: 5′ CGGGATCCATGAGACACTCAAAGAGAACTTACT 3′; CLK1-antisense: 5′ ATTTGCGGCCGC CTAGTGGTGGTGGTGGTGGTGTATACTTTTCTTCAGAAGGTC 3′. Total RNA was isolated from HUVEC using the Trizol reagent kit (Life Technologies, Grand Island, NY, USA). KAPA HiFi™ HotStart ReadyMix (2×) (KAPA BIOSYSTEMS, Boston, MA, USA) was used to amplify the double-chain DNA. The PCR product of CLK1 was cloned into the pFastBac1 vector, then transformed into *E. coli* DH5α. After the purified positive CLK1/pFastBac1 plasmid was transformed into DH10Bac *E. coli*, blue/white selection and PCR were performed to identify colonies containing the recombinant bacmid. The M13 Forward and Reverse primers were used to verify the presence of CLK1 gene in the recombinant bacmid. M13 Forward: 5′ GTTTTCCCAGTCACGAC 3′; M13 Reverse: 5′ CAGGAAAC AGCTATGAC 3′.

### 3.5. Preparation and Purification of Recombinant Human His-CLK1

Sf9 cells grown in 75 cm^2^ plate with a concentration of 2 × 10^6^ cells/mL were infected by P2 stock. The cells were harvested at 72 h post infection. Then the recombinant protein CLK1 with the 6 × His-tag at C-terminal was purified using Ni-NTA. The samples were identified by western blot (CLK1 polyclonal antibody: NBP1-67948, Novus Biologicals, Littleton, CO, USA).

### 3.6. Optimization of the Assay for CLK1

The kinase assay in this study utilized appropriate concentration of recombinant purified protein CLK1 in the presence of MBP as the substrate, MgCl_2_, MnCl_2_, ATP in kinase buffer (10 mM Tris, 1 mM EGTA, 100 μM sodium orthovanadate, 10 μM *p*-nitrophenyl phosphate (PNP), pH7.4) in a reaction volume of 20 μL. The reaction system were incubated at 25 °C for appropriate time. The Kinase-Glo^®^ Luminescent Kinase Assay Platform was used to measure kinase activity by quantitating the amount of ATP, the signals of which were recorded using PerkinElmer’s EnSpire Multilabel Plate Reader. All the above components were added to the plate by automatic dispenser (EDR-384S, BioTec, Tokyo, Japan). A series of parameters containing the amount of kinase (1.16–298 ng/mL), MBP (3.125–150 μg/mL), ATP (0.1–100 μM), Mg^2+^ (0.01–100 mM), Mn^2+^ (0.5–20 mM), the percentage of DMSO (0.03%–10%), reaction time and temperature were optimized respectively in order to get best performance. Z′ factor values was calculated as described [15]. TG003 is a cell-permeable dihydrobenzothiazolo compound as a potent, specific, and ATP-competitive inhibitor of Clk-family kinases. In this experiment, TG003 was first dissolved in deionized water as a stock solution, followed by ten-fold serial dilution from 100 μM to 1 nM in the reaction mixture. 

### 3.7. Rational Screening of CLK1 Inhibitors

In order to reduce the cost of large scale screening for CLK1 inhibitors and improve the drug discovery rate, a structure-based virtual screening was carried out with an in-house database of 21,758 compounds containing natural products and synthetic compounds. The crystal structure of CLK1 in complex with V25 at a resolution 8 Å obtained from the Protein Data Bank (PDB entry: 2VAG) was used as the initial 3D assay. All water molecules were deleted from 2VAG, and the binding site pocket was defined by V25. The hydrogen atoms were added to the protein which was then protonated at 300 K and pH 7 with Amber 99 Force Field. During the docking stage, the parameters including placement method, the first scoring function rescoring 1, and the number of retained poses were set to Triangle Matcher, London dG, and 30, respectively. In the refinement stage, the refinement method, the second scoring function rescoring 2, and the number of poses to keep were set to force field, London dG, and 10, respectively. Poses were generated by aligning ligand triplets of atoms on triplets of alpha spheres in a systematic way, and then rescored using the London dG scoring function.

After the docking parameters were set, the crystal pose of V25 was first re-docked into the binding-site pocket of CLK1, and the RMSD values between the docking and initial poses were calculated. All the compounds in database were processed through including removing all the inorganic counterions, adding hydrogen atoms, deprotonating strong acids, protonating strong bases, generating stereoisomers, and valid single 3D conformers by means of washing and energy minimizing in MOE, then subjected to the same docking procedure. After the docking, those compounds with docking scores less than −20 kcal/mol were chosen for further screening.

The screening was carried out in white 384-well plates (PerkinElmer, Santa Clara, CA, USA) according to the reaction conditions that had been optimized before, using CLK1 kinase present in the reaction system as positive control, while CLK1 kinase absent as negative control. TG003 as a reported CLK1 inhibitor was used as a reference compound. The inhibition percentage of the compounds against CLK1 were calculated according to the formula:

(1) $$\text{inhibition\%}=\left[\left({\text{RLU}}_{\text{sample}}-{\text{RLU}}_{\text{positive}}\right)/\left({\text{RLU}}_{\text{negative}}-{\text{RLU}}_{\text{positive}}\right)\right]\times 100\text{\%}$$

where the RLU_sample_, RLU_positive_, RLU_negative_ are the measured RLU values with treatment of test compounds, the wells free of test compounds, and that of without kinase, respectively.

### 3.8. Cytotoxicity Assay

MDCK cells (1.5 × 10^4^ per well) grown in 96-well plate for 24 h before treated with CLK1 inhibitors at the concentrations between 1 to 200 μg/mL or mock control solutions (2% DMSO) at 37 °C and 5% CO_2_ for 72 h. Proliferation of cells was measured by the MTT assay. The maximal non-cytotoxic concentration (CC_0_) was defined as the maximal concentration of the sample that did not exert a cytotoxic effect and kept more than 90% cells viable.

### 3.9. Cytopathic Effect (CPE) Reduction Assay

MDCK cells were seeded into 96-well plates at 1.5 × 10^4^ cells/well for 24 h before the experiments. To evaluate the pharmacological characteristics of the CLK1 inhibitors, three different drug administrations were investigated in our experiments. (1) Simultaneous treatment assay: serial dilutions of the CLK1 inhibitors along with the influenza virus H1N1 (MOI 0.01) were added to the cells. The cell viability was tested 24 h later after removal of the supernatant; (2) Pre-treatment assay: H1N1 (MOI 0.01) was pre-incubated with serial dilutions of the CLK1 inhibitors for 2 h at 37 °C before being added to MDCK cells. After 24 h incubation, the supernatant was removed and the cell viability was tested; (3) Post-treatment assay: the influenza virus H1N1 (MOI 0.01) were incubated with cells for 2h followed by removal of the supernatant instead of serial dilutions of CLK1 inhibitors. The cell viability was tested after 24 h. Ribavirin and TG003 were set as positive compounds. The cell survival rate was assessed by the MTT method, and the final spectrophotometric data were used to calculate the IC_50_. The experiment was repeated at least three times.

### 3.10. Statistical Analysis

All data analyses were conducted with Student’s *t-*test. All data are presented as mean ± SD.

## 4. Discussion

As one of eukaryotic systems used for the expression of recombinant protein, the baculovirus system has a widespread application for recombinant protein production [16], with the initial description of human interferon expression by baculovirus or AcMNPV occurring in 1983 [17]. In this study, we first utilized a baculovirus expression system for CLK1 recombinant protein expression, which was more effective than prokaryotic expression. Homo CLK1 gene was amplified and cloned into pFastBac1 donor plasmid to allow generation of an expression construct. Then an *E. coli* host strain, DH10Bac, containing a baculovirus shuttle vector and a helper plasmid, was used to generate a recombinant bacmid after transposition of the pFastBac expression construct. As a result, enough recombinant protein His_6_-CLK1 was harvested and purified by Ni-NTA column, allowing the establishment of screening system for CLK1 inhibitors discovery. 

We also found that Kinase-Glo^®^ assay showed excellent performance as a screening assay for discovery of CLK1 inhibitors. In the luminescent kinase assay, kinase activity was inversely proportional to the amount of ATP remaining in solution after a kinase reaction. In our study, a reaction system with a total volume of 20 μL containing kinase, substrate, ATP and metal ions was used to initiate the kinase reaction, and equal volume of Kinase-Glo^®^ Reagent was added followed by a kinase reaction for luminescence reading. Compared with the radioactive method in Menegay’s work [18] (Table 2), the Kinase-Glo^®^ assay, as an indirect measurement of kinase activity, which does not require isotope-labelled components, is more efficient, safe and easy to control.

Rational screening can reduce the cost and improve the drug discovery rate. According to this principle, we carried out virtual screening of 21,758 compounds, and 380 compounds with better binding properties were screened using the assay built above. Eventually, 15 compounds were found. Two tanning compound, J10688 (clypearin) and J12098 (corilagin), were found to exhibit high CLK1 inhibitory activities, with IC_50_ value of 1.2 μg/mL and 0.38 μg/mL, respectively. Further study demonstrated that during the time-of-addition assays, the EC_50s_ of clypearin and corilagin were less than 20 μg/mL, which reflected antiviral effects no matter the pre-incubation treatment, simultaneous treatment or post-treatment assays. J14848 (pinosylvin) was a stilbene compound with an IC_50_ of 83.83 μg/mL for CLK1. In the CPE reduction assay, pinosylvin showed great anti-influenza virus effects, indicating that it might be a multi-target compound. Pinosylvin and luteolin had toxic effects above 10 μg/mL, and the EC_50s_ were close to the toxic concentrations. The reason for that may be that their effective ranges were narrow.

**Table 2:** Comparison of the kinase reaction components in our experiment with Harry J.’s.

Kinase Reaction System	Authors’ Experiment	Menegay’s Experiment
recombinant CLK1	2.98 ng in 20 μL	0.5 μg in 50μL
myelin basic protein(MBP)	1 μg in 20 μL	1 μg in 50 μL
MgCl_2_	10 mM	10 mM
MnCl_2_	1 mM	2 mM
ATP	1 μM	10 μM ([γ-^32^P])
reaction temperature	room temperature	room temperature
reaction time	90 min	20 min

Clypearin was extracted from *Pithecellobium clypearia* Benth, which is a herbal medicine used in the treatment of respiratory tract diseases in China for many years [19]. Corilagin is a member of the tannin family which has been discovered in a number of medicinal plants such as *Phyllanthus amarusare* and *Geranium carolinianum*. Corilagin had shown an extensive pharmacological spectrum, including antihypertensive, antiatherogenic, antitumor and thrombolytic effects, and anti-HSV1 [20,21,22]. Pinosylvin was extracted from the knots of *Pinus sylvestris* and it has quite extensive pharmacological activities such as anti-inflammation, protection against oxidative stress and antitumor effects [23,24,25]. In this study, we found these three compounds were also CLK1 inhibitors and had anti-influenza virus effects. The CLK1 and CPE inhibitory efficiency together with other potential pharmacological effects make clypearin, corilagin and pinosylvin potential antiviral compounds. Besides CLK1, other isoforms of CLKs have not been discussed here. Whether those CLK1 inhibitors can also inhibit other isoforms of CLKs remains unclear. More antiviral assays, especially animal assays, should be applied to identify the antiviral activity of CLK1 inhibitors. These questions above will be the focus of our subsequent work.

Influenza viruses take advantage of the host-RNA-processing machinery to provide alternative splicing for their proteomic diversity. As a kinase governing alternative splicing in cells, CLK1 was cloned and expressed in this study. A CLK1 activity testing system was established using the Kinase-Glo^®^ assay platform. Compounds with CLK1 inhibitory effect were further evaluated for their antiviral effect at the cellular level. As a result, compounds including clypearin and corilagin targeting CLK1 showed significant inhibitory effect on H1N1 virus-infected cells, which implied that CLK1 may be a potential target for anti-influenza virus drug discovery. To provide further information for CLK1 as the target in future influenza therapeutics, the selectivity of these compounds, the *in vivo* antiviral effects and their mechanism still require deeper investigation.

## Supplementary Materials

Supplementary materials can be accessed at: http://www.mdpi.com/1420-3049/20/11/19653/s1.
