# Supplementary material for: Drug Discovery of Host CLK1 Inhibitors for Influenza Treatment
Source: Molecules. 2015 Nov 2;20(11):19735–47. doi: 10.3390/molecules201119653 (PMC6332427; doi:10.3390/molecules201119653)

molecules

MDPI

Supplementary Informations

Table S1. Details of receptor-ligand interactions in each sample.

Sample	Name	Docking Score	Receptor-Ligand Interaction [a]
J10688	Clypearin	−35.7352	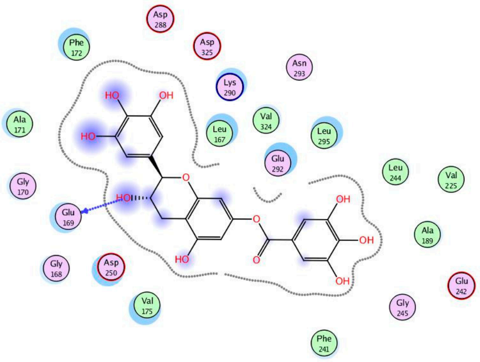
J12098	Corilagin	−36.9215	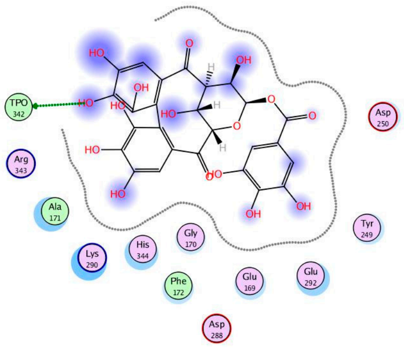
J14848	Pinosylvin	−28.9297	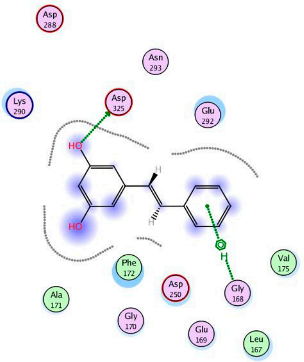
J12133	Chebulanin	−53.8421	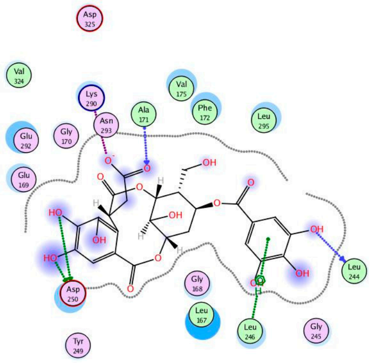

Table S1. *Cont.*

Sample	Name	Docking Score	Receptor-Ligand Interaction
J25986	Propyl Gallate	−25.2543	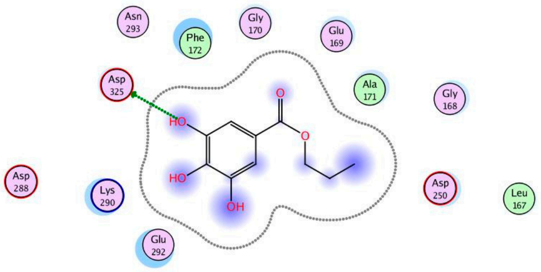
J20353	Hispidulin	−31.6968	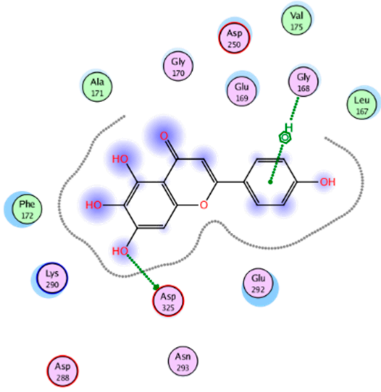
J21548	Norwogonin	−24.9415	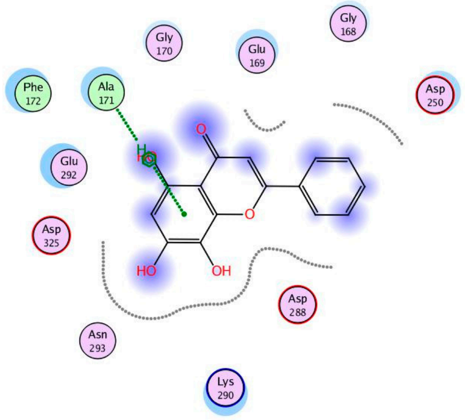
J11534	-	−39.8304	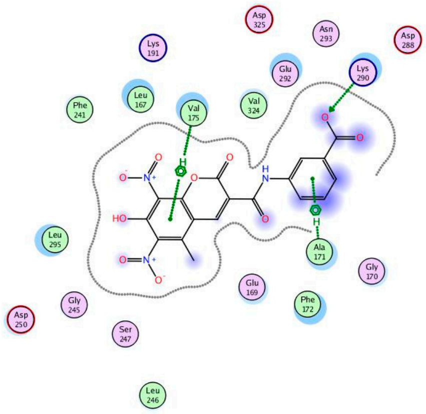

Table S1. *Cont.*

Sample	Name	Docking Score	Receptor-Ligand Interaction
J10610	Kaempferol	−27.2038	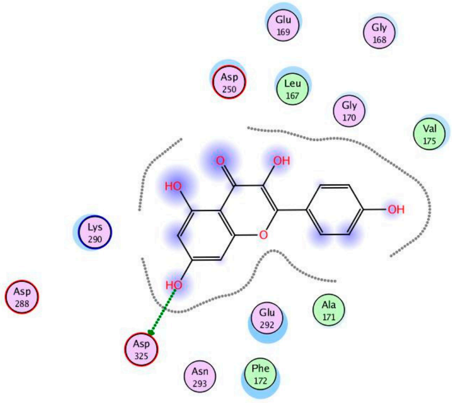
J20725	Luteolin	−29.8188	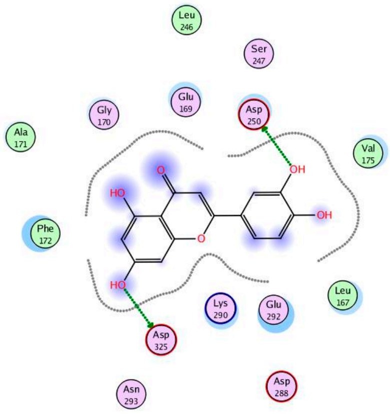
J10857	-	−30.7957	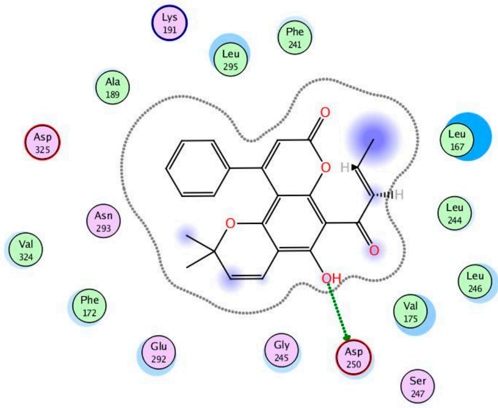
J14528	Isorhapontigenin	−30.6644	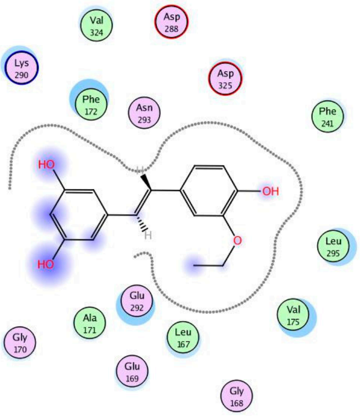

Table S1. *Cont.*

Sample	Name	Docking Score	Receptor-Ligand Interaction
J14080	Epigallocatechin gallate	−30.7234	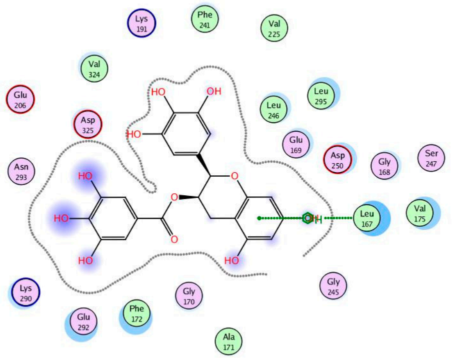
Methyl gallate	Methyl gallate	−22.3652	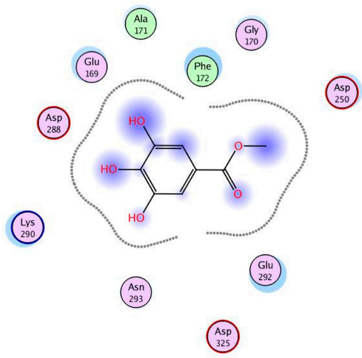
Genistein	Genistein	−23.2975	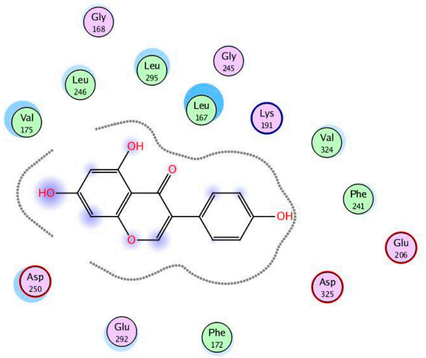

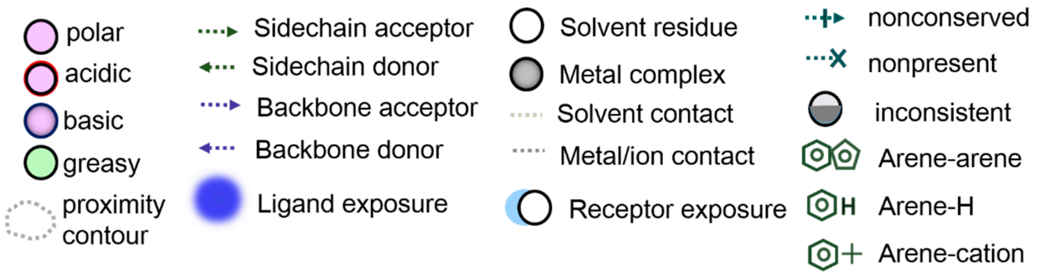

Supplement: Supplementary file 1 [file molecules-20-19653-s001.pdf]
